# Interventional Radiology in the Management of Parathyroid Disorders: Current Diagnostic and Therapeutic Approaches

**DOI:** 10.3390/jcm15093360

**Published:** 2026-04-28

**Authors:** Onur Taydas, Erbil Arik, Mehmet Ali Durmus, Volkan Tasci, Omer Faruk Topaloglu, Mustafa Ozdemir, Yusuf Ozturk, Mahmud Islam, Zulfu Bayhan, Mehmet Halil Ozturk

**Affiliations:** 1 Faculty of Medicine, Department of Radiology, Sakarya University, 54040 Sakarya, Türkiye; m.alidurmus0@gmail.com (M.A.D.); md.volkantasci@gmail.com (V.T.); ofaruktopaloglu@gmail.com (O.F.T.); drmstfrd@gmail.com (M.O.); ozturkmh@gmail.com (M.H.O.); 2 Faculty of Medicine, Department of Radiology, Marmara University, 34899 Istanbul, Türkiye; erbilarik@hotmail.com; 3 Faculty of Medicine, Department of Endocrinology, Sakarya University, 54040 Sakarya, Türkiye; yusuf_ozturk40@hotmail.com; 4 Faculty of Medicine, Department of Nephrology, Sakarya University, 54040 Sakarya, Türkiye; drisleem@gmail.com; 5 Faculty of Medicine, Department of Surgery, Sakarya University, 54040 Sakarya, Türkiye; zulfubayhan@gmail.com

**Keywords:** parathyroid, primary hyperparathyroidism, fine-needle aspiration, PTH washout, parathyroid ablation, parathyroid embolization, hydrodissection

## Abstract

This narrative review evaluates the current role of interventional radiology in the diagnosis and treatment of parathyroid disorders. In patients with biochemically confirmed hyperparathyroidism but inconclusive or discordant preoperative localization imaging, image-guided interventions can help establish a diagnosis that can alter management. This review outlines the current diagnostic and therapeutic modalities for the study of parathyroid pathology from the interventional radiologist’s perspective, highlighting novel techniques and their growing clinical adoption. The combination of ultrasound-guided fine-needle aspiration biopsy and measurement of parathyroid hormone (PTH) in the needle washout fluid can improve diagnostic specificity by providing biochemical evidence of parathyroid tissue. This is particularly useful for lesions that are difficult to differentiate from thyroid nodules or cervical lymph nodes based solely on imaging characteristics. Despite this, no widely accepted cut-off washout PTH level has yet been established. Due to the differences in assay techniques and laboratory procedures, results should be interpreted in conjunction with clinical findings and concomitant biochemical parameters. Ultrasound-guided thermal ablation techniques, especially radiofrequency and microwave ablation, have recently been reported as minimally invasive alternatives or adjuvants to surgery in appropriately selected patients. Evidence supporting parathyroid embolization is limited, but it may be considered a potential salvage option for persistent or recurrent disease. Given that most of the current evidence is derived from retrospective case series, multicenter prospective studies for technical standardization and long-term outcomes evaluation are clearly needed.

## 1. Introduction

The field of interventional radiology is rapidly evolving as novel imaging modalities and therapeutic techniques are increasingly integrated into clinical practice. Minimally invasive procedures using imaging modalities are employed not only for anatomical visualization but also for functional assessment and, in selected cases, for direct disease treatment. The widespread acceptance of such an approach has been observed across various clinical fields due to lower morbidity, shorter hospital stays, and the ability to deliver precise treatment [1].

Interventional radiology was historically perceived as an oncology-focused specialty. More recently, this specialty has expanded in its use for functional and benign disorders. Endocrine disorders, in particular, can be challenging when target lesions are small, anatomy is complex, and localization patterns are variable.

In this regard, image-guided interventional methods offer surgical alternatives or complements that reduce diagnostic uncertainty and enable more tailored therapeutic strategies [2].

Among endocrine organs, the parathyroid glands represent a particularly challenging clinical entity in terms of diagnosis and management. The small size, variable anatomical location, and potential for ectopic location could pose significant hurdles for imaging assessment and surgical planning. These features may restrict the performance of classical imaging techniques in some clinical circumstances and favor increasingly targeted diagnostic and therapeutic strategies. In fact, the current literature suggests that various image-guided interventional procedures are now gaining prominence in optimizing the management of parathyroid disease [1,2].

Interventional radiology serves not only as a diagnostic adjunct in parathyroid disease but also as a complementary modality that can provide minimally invasive therapeutic options in carefully selected patients. An integrated imaging, clinical, and interventional approach may contribute to more effective and individualized management of parathyroid disorders [1].

In this setting, ultrasound-guided fine-needle aspiration biopsy has emerged as a targeted diagnostic modality to distinguish parathyroid from thyroid disease in cervical lesions detected on imaging. In cases where definitive morphological diagnosis cannot be established on imaging alone, this minimally invasive procedure can be performed with little risk and has become a first-line interventional tool in the diagnostic process [2].

Measuring parathyroid hormone in the aspirate significantly increases the diagnostic yield of fine-needle aspiration. The strategy aims to reduce the risk of mistargeting, as imaging alone may provide limited specificity; therefore, biochemical confirmation is valuable for clinical decision-making. PTH washout analysis provides biochemical confirmation that the focus represents parathyroid tissue. In cases of ectopic lesions or in patients with a prior surgical history, this technique can play an important role in resolving diagnostic uncertainty [3].

Beyond diagnostic confirmation, interventional radiology offers thermal ablation techniques—particularly radiofrequency and microwave ablation—that enable targeted destruction of hyperfunctioning parathyroid tissue. With appropriate patient selection, these approaches can achieve satisfactory biochemical control while maintaining an acceptable safety profile [4].

Although embolization is not considered a standard therapeutic modality in parathyroid disease, it may be considered a complementary interventional option in selected, limited clinical scenarios. The principal objective of this technique is to suppress hormone production by reducing the arterial supply to hyperfunctioning parathyroid tissue, thereby offering an alternative for patients who are not suitable candidates for surgery or thermal ablation.

Available reports suggest that embolization may be employed as a palliative or bridging strategy in patients with elevated surgical risk, persistent disease despite prior interventions, or ectopically located and technically inaccessible lesions. In this respect, parathyroid embolization should be positioned not as a curative modality but rather as an adjunctive intervention aimed at reducing disease burden and achieving clinical control in carefully selected cases [5].

## 2. Methods of Literature Review

A comprehensive literature search was conducted using PubMed/MEDLINE and Scopus databases up to January 2026. The search combined the following terms: “parathyroid,” “hyperparathyroidism,” “fine-needle aspiration,” “PTH washout,” “radiofrequency ablation,” “microwave ablation,” “cryoablation,” “ethanol ablation,” “parathyroid embolization,” and “interventional radiology.” Articles published in English were considered for inclusion. Original studies, systematic reviews, meta-analyses, case series, case reports, and clinical guidelines addressing the diagnostic or therapeutic role of interventional radiology in parathyroid disease were included. Studies limited exclusively to surgical or pharmacological management were excluded.

This review aims to provide a structured overview of interventional radiological approaches in parathyroid disease, encompassing ultrasound-guided fine-needle aspiration with PTH washout analysis, thermal and non-thermal ablation modalities, and parathyroid embolization, while critically evaluating their diagnostic utility, therapeutic outcomes, and safety profiles based on the available evidence. Unlike prior reviews focusing primarily on imaging or surgical localization, this work adopts a dedicated interventional radiology perspective and uniquely integrates diagnostic and therapeutic modalities—including embolization—within a single proposed clinical algorithm.

## 3. Ultrasound-Guided Fine-Needle Aspiration of the Parathyroid Gland

Although imaging modalities can often identify a suspicious focus in parathyroid disease, the principal challenge in clinical decision-making is reliably establishing whether the detected lesion is of parathyroid origin. This uncertainty increases the risk of mistargeting and directly influences both surgical and interventional treatment planning. Current literature emphasizes that, in such scenarios, targeted diagnostic confirmation plays a pivotal role in guiding appropriate clinical management [6,7].

Ultrasound-guided fine-needle aspiration biopsy is an interventional procedure that aims to alleviate diagnostic uncertainty by directly sampling a suspicious imaging lesion. Nonetheless, the cytologic features of parathyroid tissue may substantially overlap with those of thyroid follicular lesions; therefore, cytologic assessment alone does not always permit definitive differentiation [2,8].

Ultrasound-guided fine-needle aspiration biopsy is not a routine diagnostic tool; rather, it is an adjunct reserved for specific clinical contexts. The procedure assumes special importance when there is biochemical evidence of hyperparathyroidism, but the imaging findings do not clearly identify the source of the lesion seen [9].

Additionally, fine-needle aspiration is considered for patients with discordant or negative ultrasonographic and scintigraphic findings. The procedure may thereby help establish whether the identified focus is indeed parathyroid tissue, thus limiting misdirection in treatment planning due to false-positive or false-negative imaging [10].

In patients with recurrent or persistent hyperparathyroidism, fine-needle aspiration may provide diagnostic clarification. In such cases, the procedure can help evaluate whether the new lesions are residual or recurrent parathyroid tissue, which can aid in planning reoperation or other treatment options [11].

Fine-needle aspiration is notable for its minimal invasiveness and low complication rate. Despite concerns about either fibrosis from the procedures or cellular seeding in parathyroid aspiration, available clinical evidence suggests these are rare events in everyday practice. As such, the method may be considered a safe diagnostic option in appropriately selected patients [11,12].

Several limitations, however, warrant consideration. Due to the limited discriminatory power of cytological traits in parathyroid tissue, the technique should not be considered a sole diagnostic tool, but rather an additional modality to be used in conjunction with clinical findings and imaging. In addition, poor sample collection or technically poor sampling during aspiration may limit its diagnostic value [2].

Another point of contention in the literature is the influence of fine-needle aspiration on surgical planning. Specifically, in patients considered ideal candidates for primary surgery who have already benefited from imaging for precise localization, the incremental benefit of ultrasound-guided fine-needle aspiration (FNA) may be minimal. Consequently, many authors discourage the indiscriminate use of the procedure and suggest performing it only in cases with discordant imaging and clinical findings or when diagnostic uncertainty influences therapeutic choice, rather than in all cases [9]. 

In view of these considerations, fine-needle aspiration should not be considered a standalone test in parathyroid disease; rather, when combined with imaging and clinical data, it is a tool that refines the clinical decision. When used in carefully chosen patients, it may help avoid unnecessary surgical procedures and assist in identifying which patients are most likely to benefit from further diagnostic assessment or interventional treatment strategies.

Critically appraising the existing evidence, it becomes apparent that the diagnostic value of ultrasound-guided FNA in parathyroid disease is highly context-dependent and operator-sensitive. Earlier retrospective series, such as that of Bancos et al. [13], raised concerns regarding procedural complications, including fibrosis; however, more recent studies, notably Ahmad et al. [12], have demonstrated that the risk of clinically significant fibrosis is negligible with contemporary technique. This evolution in reported safety profiles likely reflects improvements in operator expertise and patient selection over time. Furthermore, while Bakuła-Zalewska et al. [6] demonstrated a diagnostic yield for cytology alone of limited utility, the additive value of PTH washout analysis substantially improves overall diagnostic performance. Taken together, these data underscore that FNA should not be evaluated in isolation but rather as part of an integrated diagnostic pathway.

## 4. Parathyroid Hormone (PTH) Washout Analysis

Although ultrasonography is widely used in the preoperative evaluation of parathyroid lesions, their morphological characteristics often overlap with those of thyroid nodules, cervical lymph nodes, or intrathyroidal lesions. Similarly, cytological assessment based solely on fine-needle aspiration may provide limited diagnostic contribution, as the histomorphological features of parathyroid tissue can closely resemble those of thyroid-derived cells [11,14,15].

The washout technique involves measuring the parathyroid hormone (PTH) level in the fluid obtained by washing the needle and syringe with normal saline after ultrasound-guided fine-needle aspiration. Unlike cytological assessment, PTH washout is based on the direct biochemical detection of the hormone, thereby enhancing diagnostic reliability [9,16,17].

The parathyroid washout is not regarded as a routine diagnostic tool in primary hyperparathyroidism. Instead, it is classified as an adjunctive technique used in selected cases where imaging and cytological analyses are inconclusive or conflicting. When ultrasonography and Tc-99m sestamibi scintigraphy are discordant, or scintigraphy is negative, further biochemical confirmation may be required to ensure that the suspected focus is indeed parathyroid. In patients with prior surgery, accurate localization is particularly important when planning for reoperation, as potential incorrect localization may have significant clinical implications. In this context, biochemical confirmation of suspicious lesions may play a pivotal role in surgical planning [9,11,13,15,16,17].

Currently, there is no accepted cut-off value for interpreting PTH levels using the parathyroid washout technique. Significant heterogeneity in threshold values has been reported across studies, largely attributable to variations in analytical methods, sampling techniques, and laboratory protocols. The summary of these variations is shown in Table 1 [9,15,17,18,19].

Some investigators believe absolute PTH values are useful, while others argue that comparing the PTH level in washout fluid with simultaneously measured serum PTH is a better approach. A washout PTH much higher than serum PTH is strong evidence of aspirated parathyroid tissue. Despite this, it should not be used as an isolated diagnostic criterion and should be best interpreted in the context of clinical findings, serum biochemical findings, and imaging studies [9,15,16,17,18,19].

The parathyroid washout technique is considered safe when performed by an expert operator on the appropriate patient. Reported complications described in the literature mainly consist of mild adverse events such as mild localized pain, transient tenderness, and small hematomas. Infection, nerve damage, and major vascular complications have been rarely reported. Despite its favorable safety profile, the technique should be used selectively and not as a routine test without a clear clinical indication [16,18].

The washout method is occasionally objected to due to theoretical risks of parathyroid tissue seeding through the needle tract or the induction of fibrosis in the field. However, modern studies indicate clinically significant parathyromatosis or fibrotic alterations complicating further surgery are highly rare. To summarize, parathyroid washout cannot be considered a standalone diagnostic test but rather a complementary tool that aids surgical planning. Safe use is closely related to proper indication, selective use, and operator experience [9,11,13].

In summary, washout analysis is generally not required when imaging modalities show concordant findings and typical parathyroid gland localization. Nonetheless, it may be contemplated in cases of discordant ultrasonography and scintigraphy results, suspicion of an intrathyroidal parathyroid lesion, and when the differential diagnosis of cervical lesions remains elusive.

A critical assessment of the literature reveals that the primary barrier to the clinical standardization of PTH washout analysis is the absence of a universally validated cut-off value, a limitation consistently acknowledged across studies, including the comprehensive review by Obołończyk et al. [9]. The heterogeneity in reported thresholds—ranging from 65 pg/mL in Zou et al. [18] to 436.5 pg/mL in Canpolat et al. [19]—reflects not methodological inadequacy but rather fundamental differences in assay platforms and sampling techniques, as summarized in Table 1. The comparative approach advocated by Abdelghani et al. [17], wherein washout PTH is interpreted relative to simultaneous serum PTH rather than against a fixed absolute threshold, represents a pragmatic solution to this heterogeneity and has shown high positive predictive values in clinical practice. Future studies should prioritize assay harmonization and center-specific threshold validation to enable broader clinical adoption.

### Parathyroid Washout Procedure

The parathyroid washout procedure is performed in accordance with the aforementioned technical principles for ultrasound-guided fine-needle aspiration biopsy. What distinguishes the washout technique is the sampling during the post-aspiration process and the analysis of the collected material. Once the fine needle is positioned within the lesion during the procedure, aspiration is performed with minimal negative pressure or without suction. After aspiration, 0.5–1.0 mL of 0.9% normal saline is usually injected into the needle and syringe, and the aspirate is sent for parathyroid hormone assay. In this context, parathyroid washout represents an approach that differs from cytological examination based on cellular morphology, as it seeks to detect hormones biochemically in aspirated material [9,15,17,19].

## 5. Parathyroid Ablation

Surgery continues to be the treatment of choice for parathyroid adenoma and primary hyperparathyroidism (PHPT) [20]. Not every individual, however, is an appropriate candidate for surgery. This is particularly relevant for older patients, those with significant cardiopulmonary or systemic comorbidities, and patients at increased risk of complications due to previous neck surgery or recurrent disease [21]. In some situations, patient preference or concerns about surgical morbidity may also prompt consideration of alternative approaches. The use of image-guided minimally invasive treatment modalities in this patient population has gained increasing interest. The destruction of targeted parathyroid tissues can be controlled with ultrasound-guided thermal ablation techniques, particularly radiofrequency ablation. This destruction can lead to reduced serum calcium and parathyroid hormone levels, as well as decreased lesion volume [22]. Reported experiences in the literature, together with the results of recent systematic reviews, point to the applicability of these techniques as alternative or adjunctive treatments to surgery when applied with good clinical judgment and proper technical execution [23].

Ablation techniques can be broadly classified into thermal and non-thermal modalities. Thermal ablation aims to destroy target tissue by applying heat or cold. Radiofrequency ablation (RFA), microwave ablation (MWA), and cryoablation fall within this category. In RFA and MWA, controlled elevation of tissue temperature induces cellular protein denaturation and coagulative necrosis. This mechanism has supported their widespread use in the treatment of solid tumors, including hepatic and renal lesions.

In contrast, cryoablation induces tissue injury through extreme cold, leading to intracellular ice crystal formation and subsequent cellular disruption. In certain anatomical regions, tissue-preserving thermolesioning approaches may offer advantages because of the relatively greater thermal tolerance of surrounding structures [24]. The heat sink effect, commonly observed in thermal techniques such as RFA, refers to the rapid dissipation of heat along large blood vessels, which may limit ablation efficacy. Non-thermal ablation techniques destroy tissue without significant heat generation and may therefore be preferred when thermal injury is poorly tolerated or when lesions are located adjacent to vascular structures [25]. In the context of parathyroid interventions, ethanol ablation represents the principal non-thermal modality.

Ablation technique selection should be individualized based on the lesion size, location, proximity to critical structures, and anticipated therapeutic target. In particular, lesions located near the esophagus, the recurrent laryngeal nerve, or vital vascular structures require special attention to the potential for thermal complications. Ethanol ablation (non-thermal technique) is another treatment option in selected cases, especially for smaller lesions, those located more superficially, and those unfit for surgery or thermal ablation. However, because of the risk of uncontrolled ethanol diffusion and variable therapeutic effect, its use is now limited and reserved for strictly selected indications [26].

In studies evaluating ablation techniques for the treatment of parathyroid adenomas, success criteria have generally been defined within common conceptual frameworks; however, there are variations in the specific parameters used and the duration of follow-up.

In studies comparing surgery with ablation, treatment success has most often been assessed by biochemical response, with normalization of serum calcium levels as the primary endpoint. In the studies by Wei et al. and Liu et al., normocalcemia was the primary outcome measure, whereas reductions in parathyroid hormone (PTH) levels were reported as supportive biochemical parameters [27,28]. Biochemical parameters have similarly been a mainstay for outcome assessment in studies that directly compare different ablation modalities. In comparing ethanol ablation with radiofrequency ablation, Rezvanian et al. used sustained reduction in serum calcium level as the primary parameter of success. In contrast, a reduction in adenoma volume was included as a secondary assessment criterion. In a multicenter study, Wei et al. defined clinical success as a biochemical response and the absence of repeat intervention, in addition to examining differences between microwave and radiofrequency ablation [29]. Due to limited data, most definitions of outcomes in cryoablation studies are descriptive and primarily focus on technical success and short-term biochemical normalization [30].

Many studies have directly compared the two ablation techniques with surgical management. Wei et al. [27] compared microwave ablation (MWA) with parathyroidectomy for primary hyperparathyroidism and reported comparable biochemical outcomes, with MWA offering shorter procedure duration and reduced invasiveness. Similarly, Liu et al. [28] demonstrated that microwave ablation was as effective as parathyroidectomy in the short term, with a low complication rate. Eslamian et al. [31] conducted a randomized controlled trial comparing percutaneous ethanol ablation (PEA) with parathyroidectomy, reporting no significant difference between the two modalities in PTH or serum calcium control. Shorter procedure duration and shorter length of stay were reported in the PEA group [31].

On the other hand, Rezvanian et al. [32] directly compared radiofrequency ablation (RFA) with ethanol ablation, finding comparable biochemical responses between the two modalities; however, RFA was associated with better lesion control and a lower reintervention rate. Taking these data into account, ablative techniques may be a clinically relevant alternative for patients who are not candidates for surgery or who refuse surgery. Nonetheless, case-by-case evaluation of treatment is essential, taking into account lesion site, size, proximity to critical structures, and the patient’s overall clinical condition.

The literature has increasingly reported comparative analyses involving different ablation modalities. Based on studies on microwave ablation (MWA) and radiofrequency ablation (RFA) for the treatment of primary hyperparathyroidism, no single modality has been shown to be clearly superior in overall cure/success rates and safety profiles. However, studies have shown that MWA offers a shorter procedural time, and, in some subgroups, especially with large lesions, it has an advantage in complete ablation rates [29]. Cryoablation, by contrast, is supported by considerably more limited evidence in the parathyroid literature. The literature consists mainly of case reports and small case series regarding CT-guided percutaneous cryoablation of ectopic or mediastinal parathyroid adenomas. Cryoablation, as described in these reports, represents a salvage option for selected patients in whom surgical access is difficult or the risk of reoperation is considered prohibitive [33,34]. As summarized in Table 2, comparative studies consistently demonstrate that ablative techniques achieve biochemical outcomes broadly similar to parathyroidectomy, while differing in procedural time, hospitalization duration, and reintervention rates—parameters that are clinically meaningful in patient selection.

A significant amount of existing literature on parathyroid ablation reports results similar to those of surgery, though with limitations. In cohort studies comparing microwave ablation with parathyroidectomy, biochemical success rates appear indistinguishable from those of surgical management. Nevertheless, most studies are retrospective in design, and follow-up durations are generally 6–12 months [27,28]. The randomized controlled trial comparing surgery to ethanol ablation achieved randomization; however, certain methodological limitations should be considered. The study was conducted in a single center and was non-blinded. Moreover, the follow-up period for this study was 6 months. All these constitute a significant limitation with respect to long-term efficacy and generalizability [31]. Likewise, modern meta-analyses of radiofrequency ablation have shown that the biochemical response can be sustained for 12 months. Still, the available evidence base is, by and large, of retrospective studies with comparatively short follow-up periods [22]. As of now, the available evidence supports ablative techniques, but there is a clear need for prospective studies with larger patient numbers, multicenter studies, and longer follow-up.

In managing parathyroid disorders, ablative techniques should be used in an individualized approach based on patient- and lesion-related characteristics, rather than on assumptions about the absolute superiority of one treatment modality over another. Surgery is the gold standard in appropriately selected patients, but in certain situations, ablative techniques are a rational alternative or addition. Given the current literature and accrued clinical experience, ablative modalities could be considered first-line in patients who are not appropriate surgical candidates or patients in whom surgery is of higher risk. This group typically includes elderly patients with significant comorbidities, those at increased operative risk due to prior neck surgery, and patients who decline surgical intervention.

Conversely, in cases of persistent and recurrent hyperparathyroidism following surgery, ablation may offer a targeted, less invasive treatment alternative, especially when repeat surgery is technically difficult or related to higher morbidity. When one or a few adenomas can be localized by imaging, ablation represents an effective second-line option for achieving biochemical control.

While current evidence supports the feasibility and safety of these approaches, high-quality prospective data are required to establish standardized indications and long-term efficacy.

### 5.1. Parathyroid Ablation Procedure

During the procedure, patients are positioned in the supine position with slight neck extension. Neck extension enhances visualization of the spatial relationship between the parathyroid adenoma, the thyroid parenchyma, and adjacent anatomical structures, thereby facilitating probe manipulation under ultrasound guidance.

In neck thermal ablation procedures involving the thyroid or parathyroid glands, most centers perform the intervention under local anesthesia in an outpatient or day-care setting. This approach is generally incorporated into standard procedural protocols, which include technical principles such as local anesthesia, a transisthmic approach, and the moving-shot technique [35]. Several published series describe the use of hydrodissection during parathyroid RFA to protect adjacent critical structures, often with limited local lidocaine administration beyond the puncture site. These technical aspects have been detailed in a previous report [36].

All institutions have different standards for sedation. Many centers perform the procedure under local anesthesia alone; some use minimal to mild sedation for added patient comfort. In some series, the intervention has also been described as performed in operating room-like conditions under “local anesthesia plus sedation,” in selected cases, under general anesthesia [37]. In contrast, avoiding routine sedation within an awake, local-anesthesia–based approach may enhance procedural safety. In a conscious patient, the appearance of transient voice changes, dysphagia, or the sudden onset of pain or burning throughout the procedure can be an early alert. This allows quick changes or interruptions in energy delivery, reducing the risk of thermal injury to the recurrent laryngeal nerve, esophagus, or trachea. The literature on thyroid RFA implicitly suggests that intra-procedural recognition of transient voice changes may be clinically beneficial, as it allows the intervention to be adjusted to avoid permanent effects [38]. Sedation offers the advantage of improved anxiety and pain control. Nonetheless, deeper sedation levels may reduce patient input, thereby narrowing the “awake patient safety window” that allows for an initial response to alarming signals. The selection of technique and anesthetic agent should be individualized and based on the proximity of the lesion to critical structures, the extent of patient anxiety, the expected duration of the procedures, and institutional experience.

**Figure 1:**
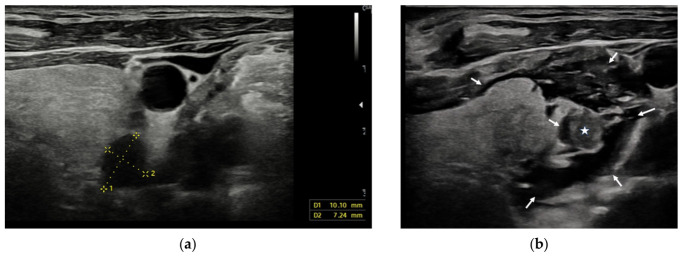
A 10 × 7 mm parathyroid adenoma located in the posteroinferior aspect of the left thyroid lobe (**a**). Following hydrodissection, a clear safety margin is observed between the parathyroid adenoma (white star) and the surrounding tissues (white arrows) (**b**).

Hydrodissection is an important technique used to prevent thermal injury during ablation by injecting fluid under ultrasound guidance between the target lesion and adjacent critical structures—particularly the recurrent laryngeal nerve, esophagus, and trachea. In radiofrequency ablation of parathyroid adenomas, the use of hydrodissection facilitates the creation of an adequate safety margin between the ablation focus and neighboring vulnerable structures. An example of appropriate hydrodissection is illustrated in Figure 1.

In the absence of adequate hydrodissection, the risk of thermal injury to the recurrent laryngeal nerve or surrounding soft tissues increases. This may not only compromise the completion of effective ablation but also result in significant neurologic complications [39].

Non-ionic fluids are most commonly preferred for hydrodissection. Due to their low electrical conductivity, 5% dextrose in water (D5W) reduces heat conduction compared with ionic crystalloids such as normal saline, thereby providing better thermal insulation within the radiofrequency energy field. Studies have demonstrated that 5% dextrose does not induce thermal tissue injury at clinically relevant distances from the energy source [40,41]. Alternatively, a more viscous colloid solution may offer additional advantages owing to its slower interstitial resorption; the separation plane created during hydrodissection may thus remain more stable over time, which is particularly beneficial when the lesion is in close proximity to critical structures such as the recurrent laryngeal nerve [40,41].

The next step after hydrodissection is inserting the ablation probe. Using ultrasound guidance, the probe is advanced in-plane and directed to the target lesion through the safest available window. When planning the trajectory of entry, the relationship of the adenoma to the tracheoesophageal groove, the thyroid capsule, and the recurrent laryngeal nerve must be carefully assessed [39,41].

**Figure 2:**
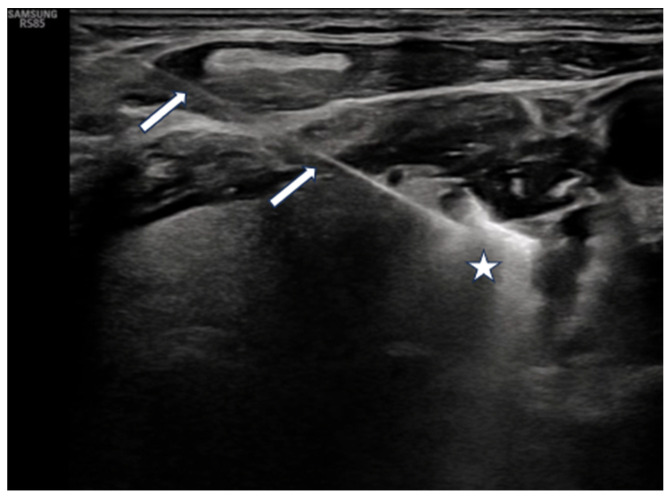
The ablation probe (white arrows) is positioned in-plane at the deepest portion of the lesion (white star). Diffuse hyperechogenicity is observed throughout the lesion, consistent with ablation-related changes.

As illustrated in Figure 2, the probe tip should be positioned as deeply as possible within the lesion under in-plane ultrasound visualization, with careful planning to allow the ablation to proceed from deep to superficial layers.

This strategy minimizes the obscuring effect of intraprocedural microbubble formation on ultrasound imaging and facilitates more controlled assessment of ablation coverage. In publications describing the moving-shot technique in parathyroid RFA, this “deep-to-superficial” approach is particularly emphasized as a key procedural principle [21,23].

Since parathyroid adenomas are usually small and located close to critical structures, the wide-amplitude moving-shot approach, commonly used for thyroid nodules, is typically modified to a more ‘micro’ approach in parathyroid interventions. In the literature, parathyroid RFA is described as a unit-by-unit, moving-shot technique, in which the lesion is conceptually divided into small ablation units. Energy delivery is initiated sequentially on each unit and continued until hyperechoic changes are noted along the entire margin of the lesion [23,42]. In this regard, two practical strategies should be adopted based on the lesion size or its proximity to critical structures. In cases of a large safety margin, employ the micro–moving–shot technique, in which probes are repositioned by 2–4 mm at controlled intervals. When the lesions are close to the recurrent laryngeal nerve (RLN)/esophagus/trachea, the probe can move less, and lesion coverage should be primarily achieved by titrating energy rather than continuous movement. In RFA, energy should be initiated at lower power and titrated gradually, based on patient tolerance and the danger to surrounding structures. Previous applications of parathyroid RFA in medical reports have used continuous RF mode with stepwise power increments in the 20–40 W range, using the moving-shot technique [35,42]. The progressive development of hyperechogenicity serves as an indirect marker of tissue heating and microbubble formation, guiding energy delivery and confirming adequate treatment margins [23].

Ultrasonography using the in-plane method is used to guide probe insertion for microwave ablation (MWA). Owing to MWA’s faster heating profile, greater weight must be given to maintaining an adequate safety margin with adjacent critical structures. Clinical studies assessing the feasibility and safety of ultrasound-guided MWA (Microwave Ablation) in primary hyperparathyroidism (PHPT) suggest that energy delivery is usually individualized according to lesion size and that the overall procedure may be short [27,43].

The procedure’s effectiveness can also be evaluated by ultrasound to determine whether the area where the ablation was performed became hyperechogenic and no longer shows vascularity. Usually, B-mode imaging and color Doppler are used for assessment. Technical success is considered confirmed if the whole lesion is included in the ablation zone. After the procedure, patients should be clinically monitored in the early period for potential complications, particularly voice changes, dysphagia, localized pain, and hematoma formation. In cases of clinical suspicion, repeat ultrasound evaluation should be performed to assess for procedure-related complications [23].

During follow-up, serum calcium and parathyroid hormone (PTH) levels should be obtained in the early post-procedure period and interpreted in conjunction with clinical findings. The key indicator of biochemical success is achieving normocalcemia, whereas changes in PTH levels are a supportive parameter [36,39].

In most patients, oral analgesics are generally sufficient for pain control, and routine antibiotics are not recommended. Patients can return to normal activities quickly but should be warned not to knock the neck area and not to exert too much energy during the first 24–48 h [44].

### 5.2. Complications and Their Management in Parathyroid Ablation

While thermal ablation procedures for parathyroid disease have shown favorable results in primary hyperparathyroidism, understanding potential adverse events and their appropriate management is a key component of procedural safety. Generally, complications are categorized as minor symptoms (pain, hematoma), major structural injuries (especially recurrent laryngeal nerve), and metabolic (hypocalcemia). Parathyroid ablation procedures have shown that overall complications are relatively low. Still, the timely recognition and management of these events are critical to optimizing clinical outcomes and ensuring the safety of the procedure [29,45].

After the procedure, patients may experience neck pain or localized discomfort, attributed to the thermal effect in the ablation zone. It is usually mild to moderate in severity. Mild symptoms are usually treated conservatively with oral analgesics and rest, as needed. Most studies report that these symptoms resolve spontaneously and rarely require prolonged intervention [36].

Even though ultrasound-guided ablation is minimally invasive, neck edema or hematoma may still occur as a result of needle passage and minor vascular injury. Most of the time, conservative management, such as local cold application, short-term watchful surveillance, and repeat ultrasound when indicated, is sufficient. Early identification of an expanding hematoma is essential to prevent potentially serious airway-related complications [36].

Hoarseness caused by recurrent laryngeal nerve (RLN) injury is one of the most serious complications of thermal ablation. This risk increases due to the thermal spread of ablation energy towards the nerve in the tracheoesophageal groove. Hence, it is recommended to perform continuous ultrasound monitoring and keep the communication line open with the patient during the procedure to detect voice changes in real time. Most reported instances of voice alteration have been transient, lasting no more than months; only rare permanent voice changes have been noted [45,46].

Heat may, but rarely, propagate around the probe during thermal ablation. This has the potential to cause superficial burns or skin injuries. In the literature, such complications are rare and, when they occur, are usually manageable with wound care and close follow-up. Minimizing this risk requires careful planning of the probe position before the procedure and careful adjustment of the energy settings [36].

A rapid drop in PTH after thermal ablation can lead to hypocalcemia, which can present clinically with paresthesia, muscle cramps, or other neuromuscular complaints. The risk of hypocalcemia should be assessed in the early post-operative period through serial testing of serum calcium and PTH levels. Management generally consists of oral calcium supplementation and, when indicated, vitamin D therapy [22].

Complications can be reduced through adequate planning, appropriate patient selection, and a meticulous technical approach. To reduce the risk of recurrent laryngeal nerve (RLN) injury, expert statements recommend creating a fluid barrier between the target lesion and adjacent critical structures with hydrodissection. During the ablation procedure, once energy delivery commences at low power, continuous ultrasound monitoring is maintained, and probe manipulation is performed with care. The micro-moving-shot procedure further lowers injury risk, in which ablations are performed unit-by-unit to a limited extent [47].

Overall, parathyroid thermal ablation carries a favorable safety profile. Minor complications are self-limited and managed conservatively, while major complications are rare and largely preventable with appropriate technique and patient selection. When they do occur, timely recognition and intervention are generally sufficient to achieve resolution. Adherence to evidence-based safety principles is essential to maintain the favorable risk–benefit profile of parathyroid ablation in contemporary clinical practice [48].

## 6. Parathyroid Embolization

Parathyroid embolization is a minimally invasive interventional radiology procedure that reduces PTH production by selectively or superselectively occluding the arterial supply of the hyperfunctioning parathyroid gland. While surgical parathyroidectomy represents the established first-line treatment, embolization may be considered in selected patients with persistent or recurrent hyperparathyroidism whose surgical options are limited or who are at elevated operative risk. A report suggested that serum calcium and PTH levels can be normalized by selectively embolizing the mediastinal adenoma [5].

Parathyroid embolization is not recommended as a routine treatment for hyperparathyroidism; it should be performed within a patient-based, multidisciplinary approach. The literature indicates that embolization is most often discussed for persistent or recurrent primary hyperparathyroidism, especially where there is a high risk of surgical complications or technically difficult reoperation. In patients with prior neck surgeries, fibrosis, anatomic distortion, and a risk of recurrent laryngeal nerve injury may warrant non-surgical therapy [20].

Reliable localization of the hyperfunctioning parathyroid gland is the most important criterion for patient selection. In cases where the target gland cannot be visualized by ultrasound, sestamibi SPECT/CT, or, if necessary, CT or MRI, embolization is not recommended. The success of the embolization depends entirely on the massive devascularization of the target gland’s arterial supply. Studies have shown that the anatomical definition of the disease and a strong single-gland component have better biochemical response rates [49,50].

A careful analysis of embolization versus ablative techniques will aid in clinical decision-making. In today’s interventional practice, percutaneous thermal ablation (RFA, MWA, laser) is typically viewed as more predictable and controllable than embolization. This is especially true for patients with single-gland disease, which can be safely targeted under ultrasound. Thus, in most centers, embolization is considered a second-line treatment for patients who are not surgical candidates and is used in select cases when ablation has failed, is technically not possible, or is more anatomically suited to embolization [5].

Parathyroid embolization’s greatest advantage is that it allows access to the blood supply of the hyperfunctioning gland without general anesthesia or surgical dissection. In persistent or recurrent disease, or when the lesion is ectopically located—such as in the mediastinal region—where reoperation is technically demanding or carries elevated risk, embolization may be considered a minimally invasive salvage option capable of achieving biochemical control in selected cases. Both early experiences provide supportive evidence for this approach, and more recent case reports that have shown selective catheterization of feeding arteries can induce clinical and biochemical response [5,51].

The key limitation of this technique, however, is that the current evidence base consists largely of case reports and small series rather than high-level studies such as randomized controlled trials or large prospective cohorts. There are several issues that arise in this context: (i) the success rates may be highly dependent on experience of the center, skill of the operator and selection of the patient; (ii) the long-term efficacies and recurrence rates cannot be compared consistently; (iii) there is no standardization in the embolic agents used, technique used and optimal targeting strategy employed. In addition, because of collateral circulation and the target gland’s vascular anatomy, technical success does not always translate into sustained biochemical control, as embolization effectiveness is directly affected by these factors. The reports today focus mainly on selected cases, describing embolization’s role in the treatment algorithm as reserved for selected patients when other options are not suitable [52].

Based on the available literature, parathyroid embolization should be proposed as a treatment option for selected patients in whom surgery or percutaneous ablation is not feasible or has failed, and in whom the hyperfunctioning gland and its feeding vascular anatomy are clear. Success depends not only on patient selection but also on careful technique planning and execution. Familiarity with the technical aspects of embolization is therefore essential to minimize procedural failure and ensure patient safety. In the following section, we will review the technical approaches used in parathyroid embolization, the vascular-targeting strategies that can be employed, and the embolic materials reported in the literature.

Compared with thermal ablation, parathyroid embolization occupies a distinctly narrower clinical niche, as reflected in the comparative scarcity of published data. While ablation studies now include randomized controlled trials and multicenter retrospective cohorts, the embolization literature remains confined to case reports and small series, with the earliest descriptions dating to Marx et al. in 1974 [51]. This temporal disparity in evidence maturation has direct implications for clinical positioning: embolization cannot be considered an equivalent alternative to ablation but rather a complementary modality for anatomically or clinically challenging scenarios. The reported success of selective arterial embolization in mediastinal adenomas—as in the case described by Yuce and Seyrek [5]—illustrates the unique access advantage of endovascular techniques in locations inaccessible to percutaneous approaches. Nonetheless, the lack of standardized embolic agents and the variable technical success across centers preclude definitive recommendations at this time.

### 6.1. Parathyroid Embolization Procedure

Parathyroid embolization aims to achieve the most complete and safest possible disruption of the arterial supply to the hyperfunctioning gland. Most clinical effectiveness can be expected when the target gland is accurately localized, the feeding artery is safely and superselectively catheterized, and adequate devascularization is achieved with the embolic agent of choice. The technical details of a procedure not only determine whether it will be successful but also relate to complications such as non-target embolization.

The parathyroid glands are usually supplied by branches of the inferior thyroid artery (ITA); they may also receive supply from the superior thyroid artery (STA), the thyroidea ima artery, or, much less commonly, mediastinal branches like the internal mammary artery. The variable vascular anatomy necessitates careful evaluation of pre-procedural imaging findings and accurate identification of feeding arteries during the procedure. According to the literature, poor vascular targeting, often exacerbated by missed collateral circulation, is one of the most common causes of technical failure in parathyroid embolization [50].

Parathyroid embolization is usually performed via the femoral or radial artery. During the procedure, the brachiocephalic trunk, subclavian artery, and the principal branches supplying the thyroid are first assessed. Selective catheterization of the artery supplying the target parathyroid gland using a microcatheter may be performed after visualization of the major vessels with standard diagnostic catheters. This method improves the effectiveness of embolization whilst reducing the risk of non-target embolization [53].

**Figure 3:**
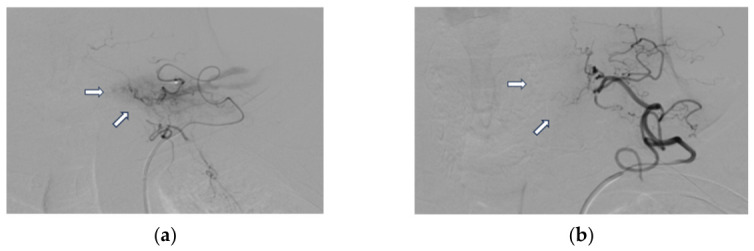
Pathological parenchymal staining arising from the left inferior thyroid artery prior to embolization ((**a**), white arrows). Resolution of the pathological staining following particle embolization ((**b**), white arrows). These images are original cases from the authors’ institution, obtained as part of routine clinical practice. Informed consent was waived in accordance with institutional policy for retrospective use of de-identified clinical images.

The patient shown in Figure 3 underwent embolization for the third time after a previously ablated lesion in the posteroinferior left thyroid lobe. A microcatheter was used to superselectively catheterize the left inferior thyroid artery, which showed pathological parenchymal staining. Following the procedure, the abnormal parenchymal staining resolved. Post-embolization PTH levels normalized.

The embolic material should be selected based on the targeted vessel diameter, the presence of collateral vessels, and the desired degree of devascularization. The literature primarily describes polyvinyl alcohol (PVA) particles and calibrated microspheres as embolic agents. It is advantageous to use particulate embolic agents, as they allow controlled, gradual embolization. While NBCA or coils have been used in selected cases to achieve more permanent devascularization, these agents carry a risk of non-target embolization. They should be used only at experienced centers. The controlled injection of particulate embolic agents until adequate parenchymal stasis is achieved is the general approach [5,52].

Technical success means the angiographic stasis in the arteries supplying the target parathyroid gland with a significant decrease in the glandular contrast enhancement. The first indicator of biochemical response is an early drop in postoperative serum PTH and calcium levels [5,53].

In summary, the technical demands of parathyroid embolization are primarily determined by accurate vascular mapping and the ability to achieve superselective catheterization. Careful selection of embolic material and meticulous procedural planning are essential to maximize clinical effectiveness and minimize complication risk.

### 6.2. Complications and Their Management in Parathyroid Embolization

Despite being minimally invasive, the complication rate of parathyroid embolization largely hinges on the embolic agent, the catheterization level, and the precision of vascular targeting. Available evidence suggests that complications are predominantly minor medical issues, but major complications, though rare, may have clinically significant morbidity.

The most common minor complication reported is transient neck pain and local tenderness due to embolization of the parathyroid tissue and the adjacent soft tissues. Discomfort frequently arises within 24–72 h after the procedure, and it readily responds to NSAIDs and/or short-term oral analgesics. This discomfort typically resolves spontaneously without long-term sequelae [5].

A sudden drop in parathyroid function can cause short-lived hypocalcemia in patients. This condition occurs more frequently when pre-procedural PTH levels are elevated and can usually be managed with oral calcium supplementation and vitamin D [50].

After satisfactory advances in superselective catheterization, another important step is the proper choice of the particulate embolic agent and size. One report on selective arterial embolization of a mediastinal parathyroid adenoma describes the use of 250–355 µm polyvinyl alcohol (PVA) particles, with marked reductions in serum calcium and PTH occurring post-embolization [5].

The literature reveals that although large series specific to parathyroid embolization are scarce, the most commonly used particulate agents in neck-region embolizations are polyvinyl alcohol (PVA) particles and calibrated microspheres, particularly in thyroid artery embolization. PVA particles are not spherical, leading to a much more heterogeneous distribution in the intravascular space. They are effective for proximal-to-mid-level occlusion, especially during selective or superselective embolization. Accordingly, the PVA should be viewed as a more suitable agent in tissues such as the parathyroid gland, which has a small vascular network and a rich collateral supply, when the risk of distal non-target migration is to be minimized.

Calibrated microspheres are considered to have predictable distal penetration due to their size; however, this size may also cause unintended distal collateral embolization. As a result, studies have reported that very small particles (<150 µm) can potentially enter distal collaterals and expose patients to the risk of non-target ischemia.

In this regard, it is acknowledged that the choice of embolic material in parathyroid embolization must be personalized not only by particle type but also by target vessel caliber, collateral circulation, and success of superselective catheterization [54,55].

Among the major complications, the most serious is non-target embolization. If embolic material is not chosen appropriately (for instance, very small particles or uncontrolled liquid embolics) or the injection rate is not right or the injection is done from a nonsuperselective position, there is an increased chance of inadvertent embolization to adjacent thyroid parenchyma, laryngeal branches, or other collateral vascular beds.

In conclusion, embolization should always be performed from a superselective location using embolics of suitable size and a controlled injection speed, as non-target embolization can result in serious morbidity [5,54].

## 7. Proposed Clinical Algorithm for the Integration of Interventional Radiology in Parathyroid Disease

The management of parathyroid disease requires a comprehensive and integrated evaluation of multiple clinical factors. Biochemical findings, imaging results, and patient- and surgery-related considerations should be assessed collectively to guide decision-making. In cases of discordant imaging, persistent or progressive disease, or increased surgical risk, interventional radiology may represent a valuable component of the diagnostic and therapeutic strategy. It must be emphasized that the current evidence base for most interventional modalities remains limited to retrospective series and case reports; accordingly, the proposed algorithm should be interpreted as a structured framework for clinical reasoning rather than a prescriptive protocol.

**Figure 4:**
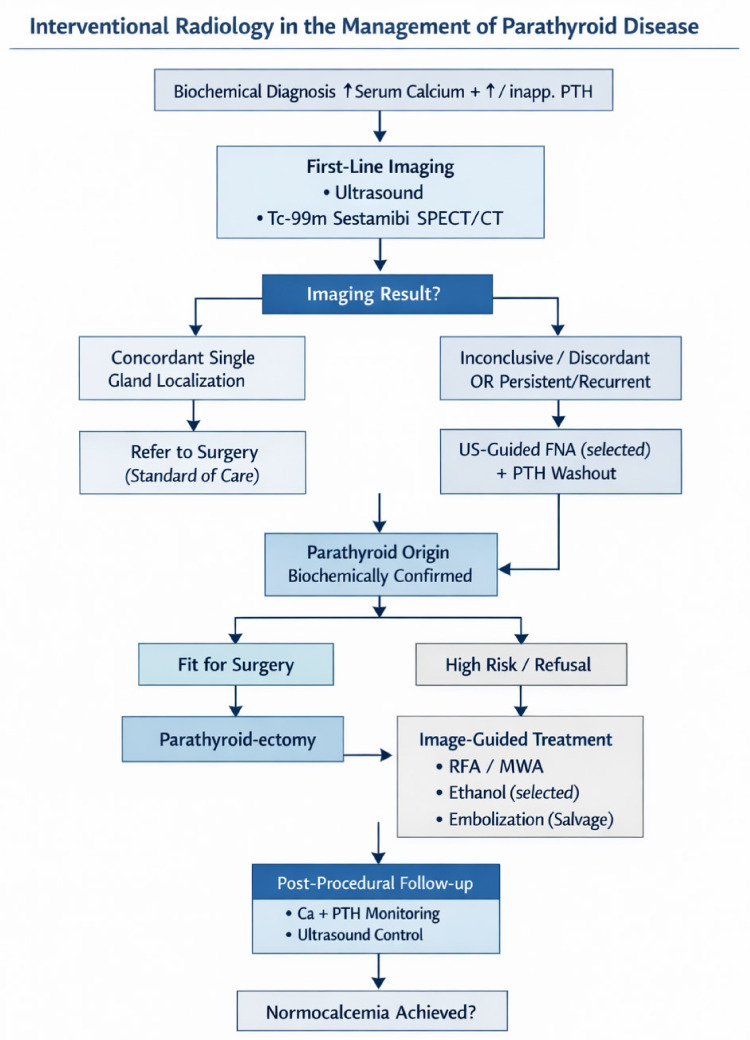
The proposal outlined a clinical workflow that integrates interventional radiology into the treatment of parathyroid disease. Interventional techniques help patients with inconclusive imaging, persistent or recurrent disease, or high surgical risk.

The proposed algorithm, illustrated in Figure 4, stratifies patients along four key dimensions: (1) surgical candidacy—distinguishing routine operative candidates, in whom surgery remains the standard of care, from high-risk or surgically ineligible patients in whom interventional alternatives may be considered; (2) disease extent—differentiating single-gland disease, which is more amenable to targeted ablation, from multigland disease, which carries a higher risk of incomplete treatment with percutaneous approaches; (3) disease course—separating primary from recurrent or persistent hyperparathyroidism, the latter group representing the most established indication for interventional techniques; and (4) evidence level—clearly demarcating established diagnostic adjuncts (FNA with PTH washout) from techniques with emerging evidence (thermal ablation) and investigational or salvage options (embolization). This stratification is intended to prevent the overextension of interventional techniques into clinical scenarios where surgical outcomes remain superior and better supported by evidence.

## 8. Conclusions

Interventional radiology offers a clinically meaningful and increasingly utilized set of diagnostic and therapeutic tools for the management of parathyroid disorders. Ultrasound-guided fine-needle aspiration combined with PTH washout analysis provides biochemical confirmation in cases of discordant or inconclusive imaging, though the absence of a universally accepted cut-off value remains an unresolved limitation requiring assay harmonization. Among therapeutic modalities, thermal ablation—particularly RFA and MWA—has demonstrated biochemical outcomes comparable to parathyroidectomy in appropriately selected patients, with MWA offering shorter procedural times and RFA associated with lower reintervention rates in head-to-head comparisons. Parathyroid embolization, while limited by a predominantly case-report-level evidence base, represents a viable salvage option when both surgery and percutaneous ablation are not feasible, particularly in ectopic or recurrent disease settings. The proposed clinical algorithm presented in this review integrates these modalities within a structured, evidence-informed decision-making framework. Nonetheless, the current body of evidence is predominantly retrospective, involves relatively small patient cohorts, and rarely extends follow-up beyond 12 months. Multicenter prospective studies with standardized outcome definitions, validated patient selection criteria, and long-term safety data are clearly required. Until such evidence becomes available, image-guided interventional techniques should be regarded as adjunctive or salvage options in carefully selected patients rather than as established alternatives to surgery, and their integration into clinical practice should proceed with appropriate caution.

In summary, surgery remains the treatment of choice for eligible patients with primary hyperparathyroidism; thermal ablation represents a reasonable alternative for those who are not surgical candidates or decline surgery, particularly in single-gland disease; and embolization should be reserved as a salvage option for anatomically inaccessible or recurrent disease when both surgery and percutaneous ablation have failed or are not feasible.

## Figures and Tables

**Table 1:** Reported PTH Cut-off Approaches for Parathyroid Washout in the Literature.

Study	Year	No. of Patients	Clinical Context	Disease Subtype	Measurement Approach	Cut-off/Criterion	Sensitivity/Specificity	Complication Rate	Key Message
Abdelghani et al. [17]	2013	45	Suspicious cervical lesions (parathyroid vs. thyroid/lymph node)	Primary HPTH, discordant imaging	Comparative with serum PTH	Washout PTH > simultaneous serum PTH	High PPV (>95% reported)	Not reported	Serum-based comparison may be more reliable than a fixed absolute threshold
Güneş et al. [15]	2023	68	Lesions difficult to differentiate on imaging	Primary HPTH	Distribution of absolute values	Markedly elevated in the positive group, very low in the negative group	ROC-based analysis; no single definitive threshold proposed	Not reported	Interpretation should emphasize clinical context and value distribution
Canpolat et al. [19]	2018	54	Primary hyperparathyroidism, US-suspicious lesions	Primary HPTH	Absolute cut-off	≥436.5 pg/mL	Sensitivity 92%—Specificity 88%	Not reported	Numerical threshold feasible but likely center-specific
Zou et al. [18]	2020	40	FNA with rapid PTH assay	Mixed (thyroidectomy patients)	Absolute cut-off	≥65 pg/mL	Sensitivity 95%—Specificity 90%	Not reported	Assay methodology significantly influences cut-off values
Obołończyk et al. (Review) [9]	2022	—	Multicenter literature	Primary HPTH (review)	Review	No universal cut-off	—	Low; mainly mild adverse events (pain, tenderness, hematoma); major complications rare	Heterogeneity is unavoidable; lack of standardization persists

**Table 2:** Comparative Studies of Surgery and Ablation in Parathyroid Adenomas and Head-to-Head Comparisons of Ablative Techniques.

Study	Design	Sample Size (n)	Compared Modalities	Disease Subtype	Follow-up	Technical Success Definition	Biochemical Cure Definition	Main Outcomes	Complication Rate/Reintervention Rate	Clinical Interpretation/Message
Wei Y et al. [27], 2022	Cohort	101	MWA vs. Parathyroidectomy	Primary HPTH	12 months	Not reported	Normocalcemia	Comparable biochemical cure rates	Not reported/Not reported	MWA is less invasive; shorter procedure duration
Liu F et al. [28], 2019	Retrospective	64	MWA vs. Parathyroidectomy	Primary HPTH	6 months	Not reported	Normalization of serum Ca and PTH	Similar reductions in serum Ca and PTH	Low complication rate (not quantified)/Not described	MWA is considered a safe alternative
Eslamian M et al. [31], 2025	Randomized controlled trial	60	Ethanol ablation vs. Parathyroidectomy	Primary HPTH, solitary adenoma	6 months	Not reported	PTH + Ca normalization	No difference in cure rates	Comparable between groups (not quantified)/Not described	Ethanol: shorter hospitalization; minimally invasive
Rezvanian P et al. [32], 2025	Retrospective	72	Ethanol ablation vs. RFA	Primary HPTH	12 months	Not reported	Sustained Ca normalization	Comparable clinical and biochemical responses	Not reported/Lower with RFA	Lower reintervention rate with RFA
Wei Y et al. [29], 2021	Multicenter retrospective	138	MWA vs. RFA	Primary HPTH	12 months	Not reported	Biochemical response + absence of repeat intervention	Similar success and complication rates	Similar between groups (not quantified)/Similar between groups	Shorter ablation time with MWA
Xu W et al. [30], 2023	Meta-analysis	12 studies	MWA vs. RFA	Primary + Secondary HPTH	Variable	Not reported	Not reported	No clear superiority demonstrated	Similar across modalities (not quantified)/Not described	Modality selection should be individualized
Meek J et al. [33], 2017	Case series	6	Cryoablation (ectopic lesions)	Ectopic (mediastinal) HPTH	9 months	Not reported	Normocalcemia	High technical success	Not reported/Not reported	Option for surgically unsuitable ectopic cases
Bayya N et al. [34], 2020	Case report	1	CT-guided cryoablation	Ectopic (mediastinal) HPTH	12 months	Not reported	Normocalcemia	Normocalcemia was achieved	Not reported	Salvage alternative in selected cases

## Data Availability

The raw data supporting the conclusions of this article will be made available by the authors on request.

## Abbreviations

The following abbreviations are used in this manuscript:

PTH	Parathyroid hormone
US	Ultrasound
RFA	Radiofrequency ablation
MWA	Microwave ablation
